# miRNA-130a Significantly Improves Accuracy of SGA Nutritional Assessment Tool in Prediction of Malnutrition and Cachexia in Radiotherapy-Treated Head and Neck Cancer Patients

**DOI:** 10.3390/cancers10090294

**Published:** 2018-08-30

**Authors:** Tomasz Powrózek, Radosław Mlak, Anna Brzozowska, Marcin Mazurek, Paweł Gołębiowski, Teresa Małecka-Massalska

**Affiliations:** 1 Department of Human Physiology, Medical University of Lublin, Radziwiłłowska 11, 20-080 Lublin, Poland; radoslawmlak@gmail.com (R.M.); marcinmazurek1212@gmail.com (M.M.); tmalecka@gmail.com (T.M.-M.); 2 Department of Oncology, Medical University of Lublin, Jaczewskiego 7, 20-090 Lublin, Poland; annabrzo@poczta.onet.pl (A.B.); pawel.golebiowski@umlub.pl (P.G.)

**Keywords:** head and neck cancer, cachexia, malnutrition, miRNA, SGA

## Abstract

Background: Investigation of novel cachexia-related markers is one of the major challenges in contemporary oncology. Among studied markers, the miRNA seems to be promising due to its possibility to regulate genes responsible for induction of inflammatory response, muscle atrophy and fat tissue wasting. The aim of the study was to investigate the role of blood-circulating miRNA-130a in prediction of cancer cachexia in 70 head and neck cancer patients (HNC) subjected to radiotherapy. Moreover, diagnostic accuracy of SGA (Subjective Global Assessment) scoring and miRNA-130a level was evaluated in various cachexia models. Results: miRNA-130a level negatively correlated with plasma TNF-α concentration (*r* = −0.560; *p* < 0.001). Patients with low miRNA expression had over 3-fold higher risk of body mass index (BMI) decrease below 18.5 after the termination of therapy; over 6-fold higher risk of losing over 5% of body weight and higher risk of >10% weight reduction odds ratio (OR) = 14.18 compared to other cases. ROC analysis performed for miRNA-130a allowed to distinguish cachectic patients (body weight loss >5%) from moderately or mildly malnourished ones with optimal sensitivity of 79.4% and specificity of 80.8% area under the curve (AUC) = 0.865). miRNA significantly improved nutritional assessment conducted using SGA, achieving the following values: sensitivity 88.6%, specificity 94.3%, positive predictive value (PPV) 93.9%, negative predictive value (NPV).89.2%. Conclusion: miRNA-130a demonstrates potential clinical utility in prediction of cachexia prior to the therapy in HNC patients. Simultaneous use of both tools—SGA and miRNA—significantly improved the accuracy in the diagnosis of cachexia.

## 1. Introduction

Cachexia is a complex wasting syndrome characterized by the reduction of fat and muscle mass, especially when it results from cancer. The following symptoms are frequently observed in cancer cachexia: reduced energy intake, significant reduction in body weight, fatigue and muscle weakness, which regards over 50% of cancer patients, including head and neck cancer patients (HNC). The above-mentioned nutritional deficits have a significant negative impact on the patients’ quality of life and contribute to the death of up to 20% of all patients [1,2,3,4]. In HNC patients, malnutrition and cachexia are frequently present at the time of diagnosis, however they may also be induced by either radical or systemic treatment (surgery, chemotherapy, radiotherapy) as side effects of the applied therapy, which further contribute to the development of the abovementioned syndromes (44–88% of HNC patients). During the disease duration, malnutrition can progress to cachexia as a result of prolonged wasting of body mass or it can be induced by treatment [5,6].

Although we are continuously gaining new knowledge about the treatment perspectives and management of patients demonstrating severe malnutrition or cachexia, the exact mechanism behind their pathophysiology is still disputable. Therefore, it is a serious challenge to predict the development of cachexia in patients at the time of diagnosis or before the application of therapy basing only on clinical factors and anthropometric examination. Most researchers accept the hypothesis of cancer cachexia as the result of tumor–host interaction leading to acute inflammatory response with overproduction of pro-inflammatory cytokines (e.g., TNF-α, IL-1, I6, and INF-γ) [7,8,9]. Recent studies revealed a crucial role of microRNA (miRNA) in the development of cancer cachexia, also in relation to the regulation of inflammatory response. These small non-coding RNA molecules can regulate gene function post-transcriptionally on the mRNA level, resulting in inhibition or promotion of protein synthesis. Therefore, miRNA are involved in the regulation of genes encoding inflammatory or muscle proteins, or agents regulating metabolism of fat tissue [10,11]. Among studied molecules, it was found that, e.g., miRNA-483, 23a, 744, 94b were altered in cachexia patients [12]. In cancer, some groups of miRNA were also identified as inductors of apoptosis of skeletal muscle cells [12,13].

According to recent studies, miRNA-130a is involved in the regulation of *TNF-α* expression; therefore, it seems to be an attractive cachexia marker related to regulation of inflammatory response [14,15]. We also confirmed this data using the bioinformatics software to predict miRNAs targeting *TNF-α.* The following software were applied for target prediction: TargetScan 7.1, miRDBase, DIANA tools, miRTar, and ceRDB-Oncomir. Among predicted miRNAs targeting *TNF-α*, all databases selected miRNA-130a as the highly conserved molecule. Based on the above, for further analysis, we selected miRNA-130a as the potential key regulator of *TNF-α* expression. In the present study we investigated the role of miRNA-130a as a predictor of cachexia in HNC patients. We also compared the accuracy of a common tool, SGA (Subjective Global Assessment) with miRNA in prediction of severe malnutrition and cachexia. Moreover, we assessed the efficacy of simultaneous usage of both these tools in cachexia prediction. 

## 2. Materials and Methods

### 2.1. Study Group

We enrolled 70 patients with histologically confirmed HNC in the study group. Patients were recruited at the Department of Oncology, Medical University of Lublin between 2014 and 2015. In all patients (median age: 63 ± 8.97 years; 80% male) radiotherapy-based (RTH) treatment was introduced. IMRT (intensity-modulated radiotherapy) technique (daily dose of 2 Gy) with the use of ONCOR (Siemens) linear accelerator was applied to perform radical RTH. To evaluate the disease stage, the TNM staging system (7-th edition) was used. The performance status of patients was assessed in accordance with ECOG-WHO (Eastern Cooperative Oncology Group–World Health Organization) scale. Alcohol consumption was evaluated according to ICD (International Statistical Classification of Diseases and Related Health Problems). 

### 2.2. Nutritional Assessment

To assess the nutritional status of patients before treatment, we used SGA scale. It included a detailed history of weight change, food intake, functional capacity alterations, symptoms from the gastrointestinal tract and results of the physical examination (muscle wasting, loss of body fat and the presence of ankle and sacral edema and ascites). Based on the above data, patients’ nutritional status was evaluated according to the score obtained from the SGA scale as: normal (0), mild (1+), moderate (2+) or severe (3+). For statistical analysis, we divided patients into 3 groups: A (well-nourished), B (moderately malnourished), C (severely malnourished). Furthermore, the patients were evaluated for nutritional risk according to NRS (Nutritional Risk Score, NRS 2002) scale which includes deterioration of nutritional status, the severity of the disease and the patients’ age. In our study group, cancer cachexia was confirmed according to ESPEN (European Society for Clinical Nutrition and Metabolism) guidelines (based on Fearon et al. definition) as a chronic DRM (disease-related malnutrition) defined by weight loss of at least 5% [16]. However, we also took into consideration the model including weight loss of at least 5% and, subsequently, the level of inflammatory marker (we used TNF-α instead of c-reactive protein (CRP), because we assumed that it was more specific to the inflammatory process that accompanies cancer) and subjected it to analysis. In addition, in our study group, we noted a large percentage of patients with loss of body weight over 10% (23/70 patients); therefore, we also assessed the accuracy of both tools (SGA and miRNA) in the assessment of malnutrition or cachexia followed by significant loss of body mass. We also evaluated the basic parameters related to nutritional status of patients: BMI (Body Mass Index), TP (total serum protein), ALB (albumin), TRF (transferrin), and PreALB (prealbumin) level. Each of the above described factors was tested before the commencement of therapy (I) and after the termination of therapy (VII). Detailed characteristics of the patients are summarized in Table 1.

Bioethical Commission of the Medical University of Lublin approved the study project (KE-0254/232/2014, permission date: 30 March 2014). Prior to the study all patients signed the informed consent form.

### 2.3. miRNA and ELISA

miRNA was isolated from 250 µL of plasma using miRNeasy serum/plasma kit (Qiagen, Hilden, Germany) and then reversely transcribed into complementary DNA (cDNA) by TaqMan Advanced miRNA cDNA Synthesis Kit (Thermo Fisher Scientific, Waltham, MA, USA). Amplification of cDNA was conducted in StepOnePlus Real-Time PCR device (Applied Biosystems, Foster City, CA, USA) with the use of TaqMan Fast Advanced Master Mix (Thermo Fisher Scientific, USA) and ready to use fluorescently labeled probes (TaqMan probes dedicated for circulating miRNA analysis) targeting miRNA-130a and miRNA-26a (internal control) (Thermo Fisher Scientific). All steps of miRNA analysis were conducted under the conditions of protocols provided by the manufacturer. The level of miRNA-130a expression was normalized in relation to miRNA-26a expression using ΔCt, 2^−ΔCt^ and 2^−ΔΔCt^ formula.

Plasma TNF-α level was measured using TNF alpha Human ELISA Kit Ultrasensitive (Thermo Fisher Scientific). The detection range was 0.2–32 pg/mL and the sensitivity was equal to the minimum detectable dose of this kit (<0.09 pg/mL).

### 2.4. Statistical Analysis

MedCalc software (version 12.7) was used to perform statistical analysis (MedCalc Software, Ostend, Belgium). U Mann–Whitney rank sum test was applied to compare the expression of miRNA-130a among patients with different clinical-demographic and nutritional features. Odds ratio (OR) with 95% Confidence Interval (95% CI) test was applied to assess the risk of miRNA expression impact on the nutritional status of the studied patients as well as to assess the risk of both SGA score and miRNA expression impact on the development of cachexia. Fisher’s exact test was used to examine the distribution of low level of miRNA and various SGA scores among patients assigned to different models of malnutrition and cachexia. Receiver operating curves (ROC) with area under the curve calculation (AUC) were used to assess the diagnostic accuracy (sensitivity and specificity) of miRNA-130a in the detection of cancer cachexia. In the study group, expression of miRNA-130a over the median score was considered as high, whereas its expression below median range was assessed as low (used for comparison high versus low). Results with *p* < 0.05 were considered as statistically significant.

## 3. Results

Among 70 studied cases, we detected high and low miRNA-130a expression in 33 and 37 patients (47.1% and 52.9% of the study group), respectively. Median TNF-α concentration in the study group was 9.85 ± 1.35 pg/mL.

### 3.1. miRNA-130a as a Predictor of Malnutrition and Cachexia

First, we examined the correlation between the expression of miRNA-130a and clinical-demographic features of the studied patients. We found that patients with high plasma TNF-α concentration demonstrated significantly lower miRNA expression in contrast to subjects with low TNF-α level (*p* = 0.029) (Table 2). 

Following the testing of miRNA-130a in individuals with various nutritional status, we divided patients into two subgroups: with high or low expression (Table 3). 

**Figure 1:**
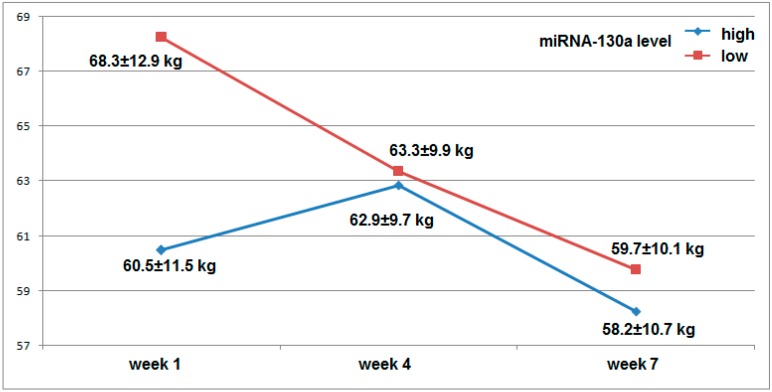
Changes in body weight during RTH in male patients with either low or high miRNA-130a expression.

Male patients who had low miRNA-130a expression level demonstrated significantly higher body weight before the commencement of therapy (week I) compared to male subjects whose miRNA expression was high (median weight: 68.3 ± 12.96 kg versus 60.5 ± 11.50 kg; *p* = 0.036). Interestingly, even though the male patients with low miRNA expression had higher body weight before the commencement of therapy (week I), they were more susceptible to weight loss during therapy (week IV and VII) in contrast to the male group with high miRNA expression (weight loss during therapy period: 11.8 ± 9.18% and 3.77 ± 4.72%, respectively; *p* = 0.0005). Dynamics of body weight loss throughout radiotherapy (week: I–IV–VII) in patients with either low or high miRNA level is presented in Figure 1.

Moreover, in the study group, low miRNA-130a level was associated with high TNF-α plasma concentration (*p* = 0.016), and miRNA level negatively correlated with TNF-α concentration (*r* = −0.560; *p* < 0.001).

We also examined the effect of miRNA-130a on the nutritional status of the studied group including separate analysis for PN (parenterally nourished) and WPN (without parenteral nutrition) patients (Table 4). 

**Figure 2:**
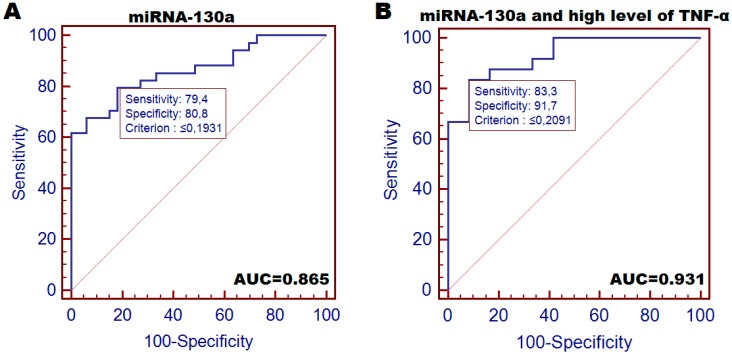
Diagnostic accuracy of plasma-circulating miRNA-130a conducted by ROC analysis with AUC calculation. (**A**) a test for distinguishing cachectic patients (loss of at least 5% of body weight) from other patients; (**B**) a test for distinguishing cachectic patients (loss of at least 5% of body weight) with subsequent high TNF-α plasma concentration from other patients.

HNC patients were at a significantly higher risk of being qualified as moderately or severely malnourished (SGA-B+C) compared to cases demonstrating high miRNA-130a expression (OR = 5.60; *p* = 0.039). Similar tendency was observed in WPN (OR = 5.526; *p* = 0.045). Moreover, patients with low miRNA expression had over 3-fold higher risk score for BMI decrease below 18.5 after the termination of therapy (VII) (*p* = 0.038); over 6-fold higher risk of over 5% loss of body weight (*p* = 0.001) and higher risk of >10% weight reduction (OR = 14.18; *p* < 0.001) in contrast to patients with high miRNA expression. ROC analysis for miRNA-130a allowed to distinguish cachectic patients (body weight loss >5%) from moderately or mildly malnourished patients with optimal sensitivity of 79.4% and specificity of 80.8% (AUC = 0.865 (0.759–0.936); *p* < 0.001) (Figure 2A). The detection of cachexia followed by high TNF-α level ROC demonstrated: 83.3% sensitivity and 91.7% specificity (AUC = 0.931 (0.794–0.988); *p* < 0.001) (Figure 2B).

### 3.2. Comparison of the Impact of SGA and miRNA-130a on the Malnutrition and Cachexia Risk Score in Various Models

The subsequent goal of the study was the comparison of subjective SGA tool with molecular factor in the prediction of malnutrition and cachexia in HNC patients. First, we compared both tools according to their influence on the risk of losing: >5% (model 1) and >10% (model 3) body mass, respectively. We also conducted an analysis taking into consideration the inflammatory marker-TNF-α. Each conducted comparison revealed significant impact of low miRNA-130a expression on high risk of losing >5% (model 2) and >10% (model 4) of body weight, respectively (OR = 8.438 and OR = 46.93, respectively). The risk score increased when it was followed by TNF-α analysis (OR = 88.0 and OR = 96.0, respectively). SGA had a significant impacton the risk of cachexia only when it was accompanied by TNF-α analysis (OR = 5.958) (Table 5).

We also compared the expression of miRNA-130a with SGA-C according to the frequency of individuals who experienced the loss of either >5% or >10% body weight. An analysis including TNF-α was also conducted (Table 6).

Significant differences between molecular marker and SGA tool regarded detection of patients who lost over 10% of body mass. miRNA detected 59.5% patients, whereas SGA 33.3% patients (*p =* 0.049). Similar analysis was conducted with the addition of SGA-B patients to the SGA-C group. Interestingly, in each analyzed model, the miRNA expression was a more reliable tool for the detection of moderate and severe malnutrition (Table 7). 

**Figure 3:**
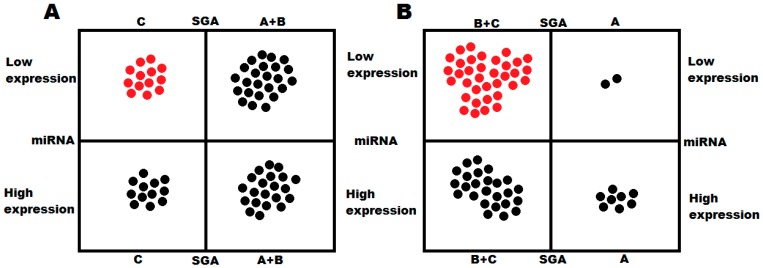
Compatibility between miRNA level and SGA scoring in the detection of. (**A**) cachexia (low miRNA-130a level and SGA-C); (**B**) moderate and severe malnutrition (low miRNA-130a level and SGA-B+C).

Comparing the results from different cachexia risk evaluation methods (SGA versus miRNA expression level), we found that low level of the studied miRNA expression was in great concordance with SGA B or C (moderate or severe malnutrition); however, this relationship was not seen when low expression was compared with SGA-C alone (Figure 3).

### 3.3. miRNA-130a Improves the Accuracy of SGA Tool in the Detection of Malnutrition and Cachexia

Finally, we examined the diagnostic accuracy of SGA and miRNA in each studied model including the loss of over 5% and 10% of body weight as well as the loss of body weight followed by high TNF-α concentration (model 1–4). The diagnostic accuracy of each tool with the calculation of negative and positive predictive value (NPV and PPV) is summarized in Table 8.

**Figure 4:**
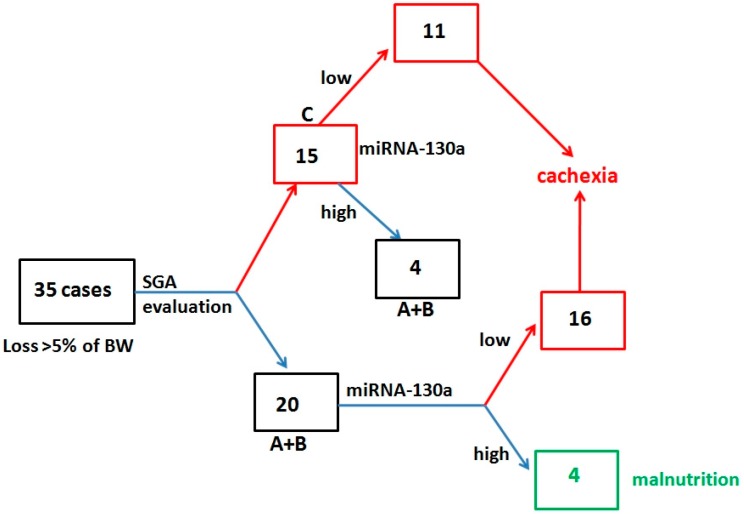
Diagnostic algorithm for cachexia and malnutrition detection followed by simultaneous evaluation of SGA and miRNA-130a.

In each proposed model, nutritional evaluation of the studied patients, conducted by simultaneous analysis of miRNA and SGA, significantly improved their diagnostic accuracy. The most significant improvement was observed especially in the NPV and PPV of the test. In the study group, among 35 patients who developed cachexia (loss of >5% body weight), 15 subjects (42.9%) were assigned to SGA-C group, and 20 underdiagnosed subjects (57.1%) were assigned to malnourished group (SGA-A+B). The additional miRNA analysis performed in the malnourished patients (SGA-A+B) allowed for the detection of 16 patients (90%) who lost at least 5% of body weight. Among 15 cases with the loss of >5% body weight, low miRNA expression was noted in 11 cases, whereas 4 had high level of miRNA. Dual nutritional evaluation followed by SGA and miRNA underdiagnosed 8 subjects (22.9%), who were classified as malnourished (Figure 4). 

### 3.4. miRNA 130a and Grade of Cachexia

In the study group 37 cases were diagnosed as cachectic (52.9% of studied cases) (diagnostic criteria-loss of 5% body weight). Using % loss of body weight (5% or 10%) we assessed utility of miRNA-130a for detection of cachexia grades. We used following criteria: grade I (loss >5% body weight), grade II (loss >10% of body weight). We found significantly lower miRNA-130a expression in patients with grade II compared to grade I of cachexia (*p* = 0.044). We also performed analysis only for the male subgroup. Similarly, we found significantly lower expression of miRNA-130a in male patients with grade II cachexia compared to grade I patients (*p* = 0.040). ROC analysis demonstrated sensitivity of 63.6% and specificity of 64.7% (AUC = 0.663; *p* < 0.05) for distinguishing grade I of cachexia from grade II. We achieved better diagnostic accuracy for distinguishing severity of cachexia in male subgroup. The ROC demonstrated 66.7% sensitivity and 70.4% specificity (AUC = 0.692; *p* < 0.05). Addiction of inflammatory marker (TNF-α) to proposed grading did not improve cachexia stratification followed by miRNA-130a, probably due to high prevalence of high TNF-α level in cachectic patients. 

### 3.5. miRNA-130a and Patients’ Survival

**Figure 5:**
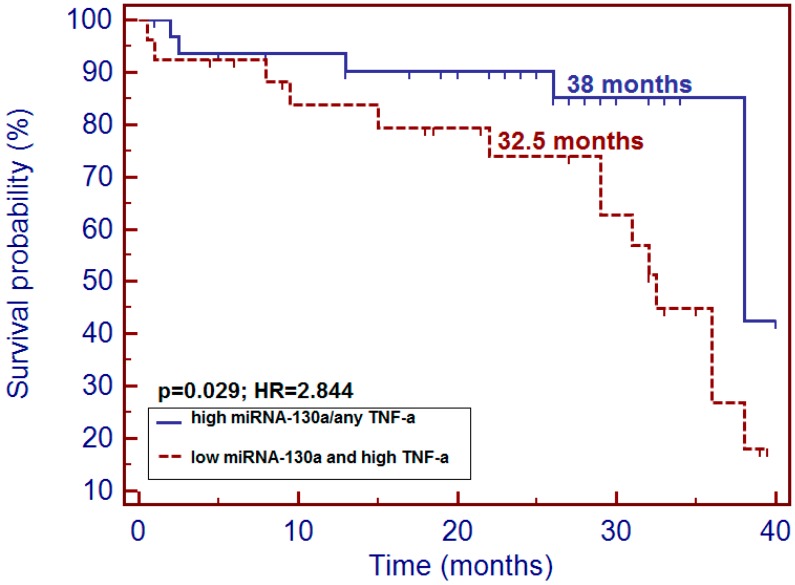
Impact of simultaneous low expression of miRNA-130a and high level of TNF-α on survival probability in the study group.

In the whole study group, patients with low miRNA-130a had insignificantly shorter overall survival (OS) compared to patients with high miRNA-130a level (32.5 versus 38 months; *p* = 0.087; hazard ratio (HR) = 2.582). Similar insignificant trend we noted in the group of male patients. However, we observed that patients with simultaneous low expression of miRNA-130a and high TNF-α level were at a higher risk of early death incidence comparing with patients demonstrating high miRNA level followed by either low or high concentration of plasma TNF-α (32.5 versus 38 months; *p* = 0.029; HR = 2.844: 95% CI (1.157–6.991) (Figure 5). We did not find correlation in miRNA-130a expression and disease recurrence in the study group (*p* = 0.136).

## 4. Discussion

Because of the heterogeneity of cachexia presentation and due to the lack of its established definition, the diagnosis of this complex wasting syndrome is often not made in a timely fashion. It seems to be predominantly the result of individual variation among patients related to their genetic predisposition. Subjective tools such as SGA or NRS followed by physical examination and lab tests are not able to predict the risk of either severe malnutrition or cachexia with satisfactory credibility. Although more accurate tools, such as CT scans, DEXA or bioimpedance are available, their accessibility and repeatability is limited [17]. Until today, several molecular markers have been tested as specific biomarkers of predisposition to cachexia. Recent studies selected candidate genes whose alterations may be closely related to the regulation of inflammatory response and wasting of either muscle or fat tissue [17,18]. Among the tested biomarkers, miRNA seems to meet the criteria of cachexia marker, defined by reliability, non-invasiveness and its key role in the regulation of genes responsible for muscle and adipocyte cell metabolism. However, only a few papers described their function in cancer cachexia in detail. 

The function of miRNA in cachexia pathophysiology is probably closely related to the regulation of systemic inflammation induced by the development of tumor in a host body [10,11,17,19]. Fabbri et al. observed an increased level of tumor-secreted miRNA-21 and 29a in lung cancer cell cultures and in murine model. Both molecules were able to bind TLR-7 and TLR-8 as ligands, which then induced TLR-mediated pro-metastatic inflammatory response and resulted in skeletal muscle atrophy [20]. In another study, Soares et al. revealed miRNA-206 and the already described miRNA-21 as promoters of muscle atrophy induced by targeting of the muscle transcription factor YY1 and the translational initiator factor eIF4E3 [21]. Also, according to Xie et al., chronic inflammatory response changes miRNA expression pattern in adipose tissue. The following miRNA variants were selected as promising candidates for studies concerning adipose tissue inflammation in cachexia-miRNA-155, 146a, 21, and 9. Interestingly, the mechanism of fat tissue loss was mediated by TLR [22]. In our study, we examined miRNA-130a as a cachexia predictor involved in the regulation of inflammatory response via targeting *TNF-α*. Recent studies confirmed strong relationship between miRNA-130a and TNF-α. Zhang et al. found that miRNA-130a directly targets and negatively regulates *TNF-α* expression in cervical cancer cell lines [15]. Similar findings were noted by Li et al., who observed negative correlation between the level of plasma TNF-α concentration and miRNA-130a expression in osteoarthritis patients [14]. Our results are in accordance with the above findings; we also found that patients with low miRNA expression had high plasma concentration of TNF-α, and the other way round (correlation *r* = −0.560; *p* < 0.001). Until today, the clinical utility of miRNA in prediction of cancer cachexia has not been fully proven. We noted, that patients with low miRNA-130a expression were at a higher risk of being classified as cachectic (in various cachexia models) compared to patients with high expression of this molecule (for weight loss >5%, OR = 8.438). When we analyzed the cachexia model with weight loss of >5% followed by high TNF-α level, the risk score significantly increased (OR = 88.0). Moreover, miRNA expression also allowed to predict the risk of significant weight loss of at least 10% of body weight (OR = 46.93 and OR = 96.0). In contrast to miRNA, SGA only had impact on the cachexia risk in inflammatory model (OR = 5.958). Moreover, ROC analysis for miRNA-130a allowed to distinguish cachectic patients (body weight loss >5%) from moderately or mildly malnourished patients with optimal sensitivity of 79.4% and specificity of 80.8% (AUC = 0.865).

According to the study results, nutritional evaluation with molecular marker (miRNA-130a) is more reliable and accurate in cachexia prediction than SGA. However, the use of both tools significantly improved diagnostic accuracy of cachexia in each studied model. For the classic cachexia definition (loss of body weight of at least 5%), the following diagnostic accuracy of both tools was achieved: sensitivity 88.6%, specificity 94.3%, PPV 93.9%, NPV 89.2%. We noted that SGA scoring missed over half of patients with cachexia and assigned them to mild or severely malnourished group (SGA-A+B). Subsequent miRNA analysis allowed to detect cachectic patients among SGA-A+B group and properly assign them to cachectic group. Dual examination conducted using both SGA and miRNA reduced the ratio of missed cases to 22.9%.

## 5. Conclusions

miRNA-130a demonstrated potential clinical utility in prediction of cachexia prior to the therapy in HNC patients. Patients with low miRNA expression are believed to be highly susceptible to cachexia, probably through the promotion of inflammatory response mediated by TNF-α. Moreover, our study revealed miRNA-130a as a significant support for SGA assessment allowing for the detection of cachectic patients missed by SGA evaluation. However, the diagnostic accuracy of miRNA-130a should be further investigated in a larger cohort of patients, and the studied marker needs scrupulous validation.

## Figures and Tables

**Table 1:** Detailed characteristics of the study group.

Factor		Study Group (*n* = 70)
Gender	Male	56 (80%)
	Female	14 (20%)
Age	median (range)	63 (42–87)
	>63	40 (57.1%)
	≤63	30 (42.9%)
Histopathological diagnosis	Squamous-cell carcinoma	64 (91.4%)
	Other	6 (8.6%)
Tumor location	Upper throat	19 (27.1%)
	- Nasopharynx	2 (2.9%)
	- Oropharynx	17 (24.2%)
	Lower throat	51 (72.9%)
	- Hypopharynx	13 (18.6%)
	- Larynx	38 (54.3%)
	Others	32 (45.7%)
T stage	T1	2 (2.9%)
	T2	9 (12.9%)
	T3	21 (30%)
	T4	38 (54.2%)
N stage	N0	21 (30%)
	N1	8 (11.4%)
	N2	36 (51.5%)
	N3	5 (7.1%)
M stage	Mx	4 (5.7%)
	M0	65 (92.9%)
	M1	1 (1.4%)
Disease stage	I	2 (2.9%)
	III	15 (21.4%)
	IVA	44 (62.9%)
	IVB	4 (5.7%)
	IVC	5 (7.1%)
Performance status (PS)	≤1	62 (89.4%)
	>1	8 (10.6%)
Type of treatment	Surgery + radiotherapy (RTH)	34 (48.5%)
	Surgery + chemoradiation	16 (22.9%)
	RTH alone	10 (14.3%)
	Induction chemoradiotherapy (CHTH) + RTH	3 (4.3%)
	Concurrent chemoradiation	7 (10%)
Alcohol consumption	Yes	29 (41.4%)
	No	41 (58.6%)
Smoking status	Smoker	57 (81.4%)
	Non-smoker	13 (18.6%)
	Current smoker	50 (87.7%)
	Former smoker	7 (12.3%)
Parenteral nutrition	Yes	12 (17.1%)
	No	58 (82.9%)
Weight (kg)	Mean ± SD	65.04 ± 12.01
Body mass index (BMI)	Mean ± SD	23.34 ± 4.55
	≥18.5	51 (72.9%)
	<18.5	19 (27.1%)
Subjective Global Assessment (SGA)	A	10 (14.3%)
	B	35 (50%)
	C	25 (35.7%)
Nutritional Risk Score (NRS)	2	47 (67.1%)
	3	20 (28.6%)
	4	2 (2.9%)
	5	1 (1.4%)
Total protein (g/L)	Median ± SD	6.71 ± 0.53
Albumin (g/L)	Median ± SD	3.34 ± 0.26
Prealbumin (g/dL)	Median ± SD	0.20 ± 0.08
Transferrin (g/L)	Median ± SD	2.50 ± 0.60

**Table 2:** Differences in miRNA-130a expression among patients with different clinical-demographic features.

Factor		miRNA-130a Expression	*p*
Gender	Male	0.211 ± 0.10	0.369
	Female	0.199 ± 0.13	
Age	<63 years	0.204 ± 0.10	0.542
	≥63 years	0.209 ± 0.11	
Histopathological diagnosis	Squamous cell carcinoma (SCC)	0.210 ± 0.11	0.09
	Other	0.153 ± 0.07	
Tumor location	Larynx	0.199 ± 0.13	0.773
	Other	0.210 ± 0.07	
Disease stage	I–III	0.188 ± 0.12	0.659
	IVA–IVC	0.206 ± 0.10	
Performance status	≤1	0.199 ± 0.11	0.218
	>1	0.220 ± 0.05	
Alcohol consumption	Yes	0.222 ± 0.11	0.392
	No	0.201 ± 0.10	
Smoking status	Smoker	0.210 ± 0.09	0.521
	Non-smoker	0.189 ± 0.13	
Parenteral nutrition	Yes	0.222 ± 0.07	0.493
	No	0.199 ± 0.11	
SGA	A	0.213 ± 0.08	0.370
	B+C	0.203 ± 0.11	
	A+B	0.196 ± 0.10	0.588
	C	0.214 ± 0.11	
Plasma TNF-α concentration	High	0.193 ± 0.07	0.029
	Low	0.222 ± 0.14	

**Table 3:** Differences in nutritional status between patients with high and low expression of miRNA-130a.

Factor (Median)	miRNA-130a Expression		*p*
	High	Low	
Weight (kg) (I) All patients	66 ± 12.30	62 ± 11.0	0.107
Weight (kg) (I) Men	60.5 ± 11.50	68.3 ± 12.90	0.036
Weight (kg) (I) Women	68 ± 7.34	67 ± 8.95	0.943
Weight (kg) (VII) All patients	58.5 ± 10.16	59 ± 9.73	0.838
Weight (kg) (VII) Men	58.2 ± 10.70	59.7 ± 10.10	0.542
Weight (kg) (VII) Women	58 ± 8.25	62 ± 7.30	0.391
BMI (I) All patients	22.95 ± 4.55	22.91 ± 4.58	0.385
BMI (I) Men	24.39 ± 4.58	22.99 ± 4.38	0.217
BMI (I) Women	22.57 ± 4.32	19.16 ± 4.36	0.296
BMI (VII) All patients	20.07 ± 3.85	19.37 ± 3.86	0.351
BMI (VII) Men	20.42 ± 3.85	19.37 ± 3.88	0.315
BMI (VII) Women	19.20 ± 3.89	18.68 ± 3.85	0.432
Transferrin (g/L)	2.50 ± 0.60	2.50 ± 0.61	0.418
Prealbumin (g/dL)	0.20 ± 0.08	0.20 ± 0.08	0.774
TP (g/L) (I)	6.71 ± 0.52	6.65 ± 0.54	0.611
TP (g/L) (VII)	6.58 ± 0.67	6.28 ± 0.68	0.101
Albumin (g/L) (I)	3.34 ± 0.42	3.34 ± 0.25	0.857
Albumin (g/L) (VII)	3.25 ± 0.40	3.09 ± 0.44	0.095
TNF-α plasma level (pg/mL)	9.41 ± 1.30	10.11 ± 1.37	0.016

**Table 4:** Impact of miRNA-130a level on the nutritional status of studied patients. (I)–measurement conducted before the commencement of therapy; (VII)–measurement conducted after the termination of therapy; (UW)–underweight; (OW)–overweight; (N)–normal.

Factor		miRNA-130a Expression		
		High	Low	*p*, OR (95% CI)
SGA All patients	A	8 (80%)	2 (20%)	0.039 5.60 (1.095–28.65)
	B and C	25 (41.7%)	35 (58.3%)	
	A and B	21 (46.7%)	24 (53.3%)	0.915 0.948 (0.356–2.523)
	C	12 (48%)	13 (52%)	
SGA Without parenteral nutrition	A	7 (77.8%)	2 (22.2%)	0.045 5.526 (1.037–29.453)
	B and C	19 (38.8%)	30 (61.2%)	
	A and B	19 (46.3%)	22 (53.7%)	0.719 1.234 (0.393–3.875)
	C	7 (41.2%)	10 (58.8%)	
SGA Parenterally nourished	A	1	0	0.591 2.539 (0.085–75.77)
	B and C	6 (54.5%)	5 (45.5%)	
	A and B	2 (50%)	2 (50%)	0.680 0.60 (0.053–6.795)
	C	5 (62.5%)	3 (37.5%)	
NRS All patients	2 and 3	33 (49.3%)	34 (50.7%)	0.211 6.797 (0.338–136.68)
	4	0	3	
	2	22 (46.8%)	25 (53.2%)	0.936 0.960 (0.354–2.607)
	3 and 4	11 (47.8%)	12 (52.2%)	
NRS Without parenteral nutrition	2 and 3	26 (46.4%)	30 (53.6%)	0.350 4.344 (0.200–94.59)
	4	0	2	
	2	18 (46.2%)	21 (53.8%)	0.771 1.179 (0.390–3.566)
	3 and 4	8 (42.1%)	11 (57.9%)	
NRS Parenterally nourished	2 and 3	7 (63.6%)	4 (36.4%)	0.355 5.00 (0.166–150.93)
	4	0	1	
	2	4 (50%)	4 (50%)	0.417 0.333 (0.023–4.736)
	3 and 4	3 (75%)	1 (25%)	
BMI (I) All patients	<24.9 (N and UW)	22 (46.8%)	25 (53.2%)	0.936 0.960 (0.354–2.607)
	>25.0 (OW)	11 (47.8%)	12 (52.2%)	
	<18.5 (UW)	10 (45.5%)	12 (54.5%)	0.848 0.906 (0.329–2.493)
	>18.5 (N and OW)	23 (47.9%)	25 (52.1%)	
BMI (I) Without parenteral nutrition	<24.9 (N and UW)	18 (46.2%)	21 (53.8%)	0.771 1.179 (0.390–3.566)
	>25.0 (OW)	8 (42.1%)	11 (57.9%)	
	<18.5 (UW)	8 (44.4%)	10 (55.6%)	0.969 0.978 (0.319–2.994)
	>18.5 (N and OW)	18 (45%)	22 (55%)	
BMI (I) Parenterally nourished	<24.9 (N and UW)	4 (50%)	4 (50%)	0.417 0.333 (0.023–4.736)
	>25.0 (OW)	3 (75%)	1 (25%)	
	<18.5 (UW)	2 (50%)	2 (50%)	0.680 0.60 (0.053–6.795)
	>18.5 (N and OW)	5 (62.5%)	3 (37.5%)	
BMI (VII) All patients	<24.9 (N and UW)	24 (45.3%)	29 (54.7%)	0.583 0.736 (0.246–2.200)
	>25.0 (OW)	9 (52.9%)	8 (47.1%)	
	<18.5 (UW)	5 (26.3%)	14 (73.7%)	0.038 3.409 (1.068–10.880)
	>18.5 (N and OW)	28 (54.9%)	23 (45.1%)	
BMI (VII) Without parenteral nutrition	<24.9 (N and UW)	20 (44.4%)	25 (55.6%)	0.545 0.739 (0.277–1.969)
	>25.0 (OW)	13 (52%)	12 (48%)	
	<18.5 (UW)	5 (31.3%)	11 (68.7%)	0.153 0.422 (0.129–1.380)
	>18.5 (N and OW)	28 (51.9%)	26 (48.1%)	
BMI VII Parenterally nourished	<24.9 (N and UW)	4 (50%)	4 (50%)	0.417 0.333 (0.023–4.636)
	>25.0 (OW)	3 (75%)	1 (25%)	
	<18.5 (UW)	0	3	0.07 0.048 (0.002–1.279)
	>18.5 (N and OW)	7 (77.8%)	2 (22.2%)	
Weight loss All patients	<%5	21 (63.6%)	12 (36.4%)	0.001 6.344 (2.206–18.24)
	>%5	8 (21.6%)	29 (78.4%)	
	<10%	27 (57.4%)	20 (42.6%)	0.001 14.18 (2.974–67.55)
	>10%	2 (8.7%)	21 (91.3%)	
Weight loss Without parenteral nutrition	<%5	28 (66.7%)	14 (33.3%)	<0.001 9.20 (2.882–29.36)
	>%5	5 (17.9%)	23 (82.1%)	
	<10%	31 (62%)	19 (38%)	<0.001 14.68 (3.059–70.48)
	>10%	2 (10%)	18 (90%)	
Weight loss Parenterally nourished	<%5	4 (80%)	1 (20%)	0.216 5.333 (0.375–75.78)
	>%5	3 (%)	4 (%)	
	<10%	7 (77.8%)	2 (22.2%)	0.070 21.0 (0.782–564.18)
	>10%	0	3	

**Table 5:** Impact of SGA scoring and miRNA expression on cachexia risk in different models.

**Model 1**		**Weight Loss >5%**	**Weight Loss <5%**	***p*, OR (95% CI)**
SGA	A+B	20 (44.4%)	25 (55.6%)	0.215 1.88 (0.695–5.061)
	C	15 (60%)	10 (40%)	
miRNA-130a	Low expression	27 (73%)	10 (27%)	<0.001 8.438 (2.873–24.78)
	High expression	8 (24.2%)	25 (75.8%)	
**Model 2**		**Weight Loss >5% + High TNF-α**	**Weight Loss <5% + Low TNF-α**	***p*, OR (95% CI)**
SGA	A+B	12 (52.2%)	11 (47.8%)	0.0395 5.958 (1.090–32.57)
	C	13 (86.7%)	2 (13.3%)	
miRNA-130a	Low expression	22 (95.7%)	1 (4.3%)	<0.001 88.0 (8.226–941.37)
	High expression	3 (20%)	12 (80%)	
**Model 3**		**Weight Loss >10%**	**Weight Loss <10%**	***p*, OR (95% CI)**
SGA	A+B	15 (32.6%)	31 (67.4%)	0.951 0.968 (0.339–2.763)
	C	8 (33.3%)	16 (66.7%)	
miRNA-130a	Low expression	22 (59.5%)	15 (40.5%)	<0.001 46.93 (5.772–381.66)
	High expression	1 (3%)	32 (97%)	
**Model 4**		**Weight Loss >10% + High TNF-α**	**Weight Loss <10% + Low TNF-α**	***p*, OR (95% CI)**
SGA	A+B	10 (40%)	15 (60%)	0.09 3.375 (0.813–14.02)
	C	9 (69.2%)	4 (30.8%)	
miRNA-130a	Low expression	18 (85.7%)	3 (14.3%)	<0.001 96.0 (9.053–1018)
	High expression	1 (5.9%)	16 (94.1%)	

**Table 6:** Comparison of distribution of SGA-C score and low miRNA expression between patients assigned to different models of cachexia.

**Model 1**		**Weight Loss >5%**	**Weight Loss <5%**	***p***
SGA	C	15 (60%)	10 (40%)	0.407
miRNA-130a	Low expression	27 (73%)	10 (27%)	
**Model 2**		**High TNF-α + Weight Loss >5%**	**Low TNF-α + Weight Loss <5%**	***p***
SGA	C	13 (86.7%)	2 (13.3%)	0.550
miRNA-130a	Low expression	22 (95.7%)	1 (4.3%)	
**Model 3**		**Weight Loss >10%**	**Weight Loss <10%**	***p***
SGA	C	8 (33.3%)	16 (66.7%)	0.049
miRNA-130a	Low expression	22 (59.5%)	15 (40.5%)	
**Model 4**		**High TNF-α + Weight Loss >10%**	**Low TNF-α + Weight Loss <10%**	***p***
SGA	C	9 (69.2%)	4 (30.8%)	0.387
miRNA-130a	Low expression	18 (85.7%)	3 (14.3%)	

**Table 7:** Comparison of distribution of SGA-B+C score and low miRNA expression between patients assigned to different models of cachexia.

**Model 1**		**Weight Loss >5%**	**Weight Loss <5%**	***p***
SGA	B+C	31 (44.3%)	29 (55.7%)	0.033
miRNA-130a	Low expression	27 (73%)	10 (27%)	
**Model 2**		**High TNF-α + Weight Loss >5%**	**Low TNF-α + Weight Loss <5%**	***p***
SGA	B+C	24 (72.7%)	9 (27.3%)	0.036
miRNA-130a	Low expression	22 (96.7%)	1 (3.3%)	
**Model 3**		**Weight Loss >10%**	**Weight Loss <10%**	***p***
SGA	B+C	20 (33.3%)	40 (66.7%)	0.034
miRNA-130a	Low expression	21 (56.8%)	16 (43.2%)	
**Model 4**		**High TNF-α + Weight Loss >10%**	**low TNF-α + Weight Loss <10%**	***p***
SGA	B+C	19 (57.6%)	14 (42.4%)	0.038
miRNA-130a	Low expression	18 (85.7%)	3 (14.3%)	

**Table 8:** Diagnostic accuracy of either miRNA or SGA alone and dual analysis of both markers in detection of cachexia in various models. (BW–body weight, PPV–positive predictive value, NPV–negative predictive value).

Cachexia Model		Sensitivity	Specificity	PPV	NPV
miRNA	Loss >5% of BW	77.1%	71.4%	73%	75.8%
SGA-C		42.9%	71.4%	60%	55.6%
SGA-C + miRNA		88.6%	94.3%	93.9%	89.2%
miRNA	high TNF-α + loss >5% of BW	88%	45%	70.7%	78.6%
SGA-C		50%	60%	61.9%	52%
SGA-C + miRNA		84.6%	90%	92.3%	90%
miRNA	Loss >10% of BW	91.3%	66%	56.8%	93.9%
SGA-C		39.1%	66%	64%	68.9%
SGA-C + miRNA		91.3%	91.5%	84%	95.6%
miRNA	high TNF-α + loss >10% of BW	94.7%	50%	58.1%	92.9%
SGA-C		47.4%	53.8%	57.1%	58.3%
SGA-C + miRNA		94.7%	84.6%	81.8%	95.6%
